# A New Italian Purple Corn Variety (Moradyn) Byproduct Extract: Antiglycative and Hypoglycemic In Vitro Activities and Preliminary Bioaccessibility Studies

**DOI:** 10.3390/molecules25081958

**Published:** 2020-04-23

**Authors:** Lucia Ferron, Raffaella Colombo, Barbara Mannucci, Adele Papetti

**Affiliations:** 1 Department of Drug Sciences, University of Pavia, Viale Taramelli 12, 27100 Pavia, Italy; lucia.ferron01@universitadipavia.it (L.F.); raffaella.colombo@unipv.it (R.C.); 2 FlaNat Research Italia Srl, Via Giuseppe di Vittorio 1, 20017 Rho (Milano), Italy; 3 Centro Grandi Strumenti, University of Pavia, Via Bassi 21, 27100 Pavia, Italy; barbara.mannucci@unipv.it

**Keywords:** *Zea mays* L., purple corn byproduct, circular economy, polyphenols, anthocyanins, antiglycative capacity, advanced glycation end products, hypoglycemic effect, in vitro simulated digestion process

## Abstract

The reuse of byproducts from agricultural and food industries represents the key factor in a circular economy, whose interest has grown in the last two decades. Thus, the extraction of bioactives from agro-industrial byproducts is a potential source of valuable molecules. The aim of this work was to investigate the in vitro capacity of byproducts from a new Italian corn variety, named Moradyn, to inhibit the accumulation of advanced glycation end products (AGEs) involved in several chronic age-related disorders. In addition, the hypoglycemic effect of Moradyn was tested by in vitro enzymatic systems. A Moradyn phytocomplex and its purified anthocyanin fraction were able to inhibit fructosamine formation and exhibited antiglycative properties when tested using BSA-sugars and BSA-methylglyoxal assays. These properties could be attributed to the polyphenols, mainly anthocyanins and flavonols, detected by RP-HPLC-DAD-ESI-MS^n^. Finally, a Moradyn phytocomplex was submitted to a simulated in vitro digestion process to study its bioaccessibility. Moradyn could be considered as a promising food ingredient in the context of typical type 2 diabetes risk factors and the study will continue in the optimization of the ideal formulation to preserve its bioactivities from digestion.

## 1. Introduction

Nowadays, the interest towards purple corn and its phytocomplex composition is rapidly growing. Pigmented maize varieties are typical Peruvian and Bolivian crops, used to prepare local drinks and desserts, whose bright color is due to the presence of high anthocyanin levels in their phytocomplex [1]. All maize varieties generally possess genes correlated to pigment synthesis, but Southern American environmental factors such as temperature, light exposure, and UV radiation elicit biosynthetic pathways leading to anthocyanin accumulation [2].

In the literature, research investigating the composition of purple corn extracts obtained from different tissues, i.e., kernel, cob, husk, and silk, has been reported [1,2,3,4,5,6]. All extracts share a common basic composition consisting of six main anthocyanins: Cyanidin-3-*O*-glucoside, perlagonidin-3-*O*-glucoside, peonidin 3-*O*-glucoside, and their malonylated forms [1,5,7,8]. Besides these major compounds, other derivatives have been detected: Catechin-(4,8)-pelargonidin-3,5-di-*O*-glucoside, afzelechin-(4,8)-pelargonidin-3,5-di-*O*-glucoside [3]; cyanidin-, peonidin-, and perlargonidin-3-*O*-di-malonylglucoside [9], cyanidin-3-*O*-succinylglucoside, cyanidin-3-*O*-malonylglucoside-5-*O*-glucoside [5], and cyanidin-3,5-di-*O*-glucoside, this one identified for the first time in purple corn by Žilić et al. [10]. Moreover, several authors have detected ethyl-malonylated anthocyanins that probably originate during the extraction process, and which are known to stabilize pigments, thus prolonging shelf life [5,8].

Husk and cob extracts are richer in anthocyanins than kernel extracts, ranging from 0.49% to 4.6% (dry weight) for cobs [1,5]; moreover, husks and cobs differ from kernels for the presence of perlagonidin in their phytocomplex [3].

Nowadays, several food byproducts such as olive leaves and oil mill wastewater, grape skin, fruit and vegetable seeds, and artichoke bracts and stems have been deeply investigated for their important role in the inhibition of advanced glycation end products (AGEs) formation [11,12,13,14,15]. AGEs are typical adducts derived from Maillard reaction or non-enzymatic glycation process. These compounds are involved in many chronic diseases, such as cardiovascular pathologies, type 2 diabetes, and neurodegenerative disorders, and their formation increases in association with hyperglycemia and metabolic dysfunctions [16,17,18,19]. Thanks to their high polyphenol content, purple corn byproducts have been associated with several health properties such as antioxidant, anti-inflammatory, and antiglycative activity [2,10,20,21,22,23]. Moreover, in vitro and in vivo studies demonstrate their capacity in the prevention of cardiovascular and type 2 diabetes risk factors by exerting a strong anti-hypertensive effect, lowering cholesterol level, ameliorating insulin resistance, and improving lipid profile in high-fat diet treated rats [24,25,26,27,28].

However, it is well known that all these health benefits can occur only if the putatively active compounds are bioavailable. Bioavailability is markedly affected by bioaccessibility, i.e., the release of compounds after their intake with food, and by the chemical transformations occurring during gastrointestinal metabolism (digestibility factor) [29,30]; therefore, the evaluation of these two parameters is mandatory in order to adequately investigate the effectiveness of bioactive molecules in vitro [31,32].

Recently, FlaNat Research Italia Srl (Rho, MI, Italy) developed a new Italian purple corn variety, named Moradyn, which is able to sprout and grow in northern Italy, maintaining a highly pigmented cob. Thus, the aim of the present work is to investigate the health properties of Moradyn phytocomplex cob extract (CE), compared with its purified anthocyanin fraction (AF). In particular, the capacity to avoid the accumulation of AGEs was evaluated and hypoglycemic effects were assessed by using in vitro assays. Furthermore, phytocomplex composition was completely characterized by LC-MS technique and a preliminary bioaccessibility study was performed by a simulated in vitro static digestion procedure.

## 2. Results and Discussion

First of all, Moradyn CE was chemically characterized and tested for its antiglycative and hypoglycemic activities, as well as its isolated AF. Then, the phytocomplex was submitted to a simulated in vitro static digestion process for the evaluation of its bioaccessibility.

### 2.1. Chemical Characterization of Moradyn Cob Extract by RP-HPLC-DAD-ESI-MS^n^

**Figure 1:**
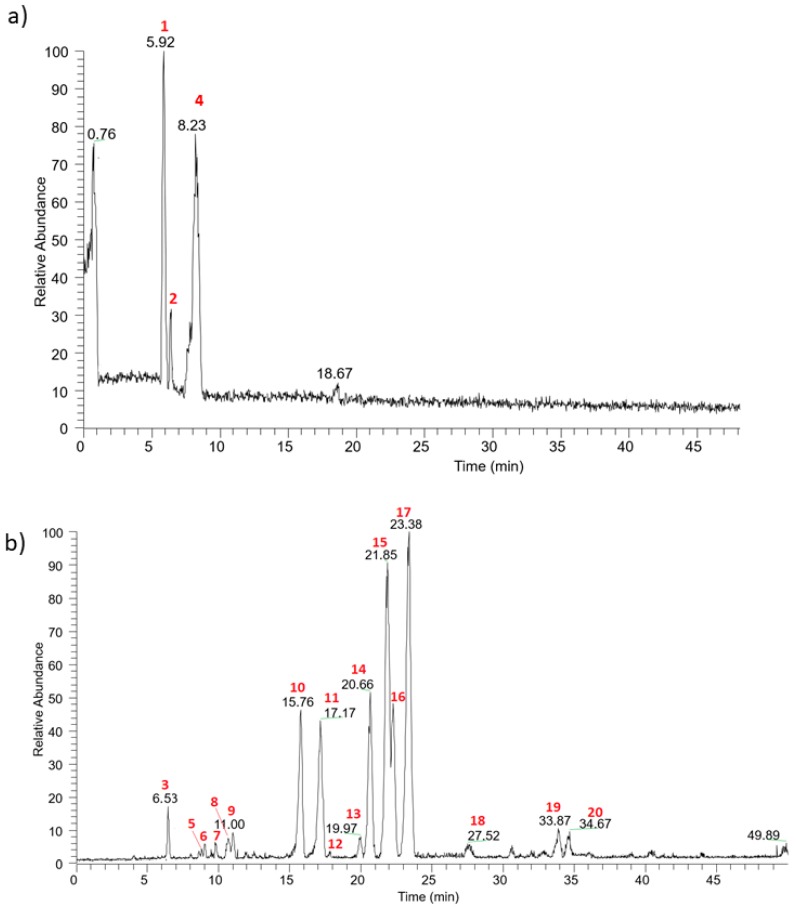
Base peak chromatograms registered in (**a**) positive and (**b**) negative ionization mode for Moradyn CE.

The qualitative profile of Moradyn CE was investigated by RP-HPLC-DAD-ESI-MS^n^ using a data-dependent acquisition mode. Figure 1a,b reports the base peak chromatogram registered in the positive and negative ionization modes, respectively. Twenty compounds were detected and identified by comparing their selectivity, UV-Vis spectra, information on pseudo-molecular ions, and fragmentation patterns with reference to pure standard compounds, where commercially available, or with data reported in the literature. Table 1 summarizes all the compounds identified in Moradyn cob extract by LC-MS^n^. 

Three anthocyanins were detected: cyanidin-3-*O*-glucoside (**1**) (*m*/*z* 449 [M^+^]), pelargonidin-3-*O*-glucoside (**2**) (*m*/*z* 433 [M^+^]), and peonidin-3-*O*-glucoside (**4**) (*m*/*z* 463 [M^+^]). All these compounds lost their hexosyl moiety (162 Da), yielding their corresponding aglycons, and their structures were confirmed by comparison with standard pure compounds tested in the same experimental conditions. Their presence has been previously reported in pericarp-pigmented corn lines from the landrace Apache Red [2], in the pericarp of cvs Arrocillo and Peruano, and in the endosperm of cv Purepecha [33]. Moreover, the same compounds have been generally identified in purple corn cob (*Zea mays* L.), seed (cv Zihei), husk [1,4,8,9,22], and also in the waste derived from a commercial purple corn colorant [6]. Cyanidin-3-*O*-glucoside was generally the main component, as confirmed by the analysis of purple corn husk deriving from 295 selected lines from the 2006 breeding population [4].

Concerning flavonoids, kaempferol and isorhamnetin derivatives differently glycosylated were the most representative. Considering the loss of 162 amu and the fact that the relative abundance of the radical aglycon ion (deriving from a hemolytic cleavage of deprotonated compound) is higher than the aglycon (deriving from the fragmentation of heterolytic cleavage) [34], compound **17** was identified as isorhamnetin-3-*O*-hexoside (*m*/*z* 477 [M − H]^−^). The structure of isorhamentin aglycon was confirmed by the presence of the intense peculiar ion at *m*/*z* 300 in MS^3^ spectrum, which led us to discriminate the isobaric compounds rhamnetin and isorhamentin [35]. Two isorhamnetin derivatives differently diglycosylated were identified: isorhamnetin-7-*O*-rutinoside (compound **15**) and isorhamnetin 3,7-di-*O*-hexoside (compound **9**). The first one (*m*/*z* 623 [M − H]^−^) fragmented producing the MS^2^ base peak at *m*/*z* 315 [M − 308]^−^ and a less intense peak ion at *m*/*z* 477 [M − 146]^−^ according to the fragmentation of a disaccharide residue with a 1→2 linkage between the monosaccharides [36]. The second one (*m*/*z* 639 [M − H]^−^) fragmented showing the sequential loss of two hexose moiety and was detected as isorhmanetin-3,7-di-*O*-glucoside. Finally, the last isorhamnetin derivative detected was compound **20**; it was putatively identified as isorhamentin-3-*O*-hexosyl-7-*O*-glucuronilhexoside by the loss of a glucuronilhexosyl moiety (338 amu) followed by the subsequent loss of another hexoside. The same fragmentation pattern was registered for a kaempferol derivative, indicating keampferol-3-*O*-hexosyl-7-*O*-glucuronilhexoside as the putative chemical structure (compound **19**, *m*/*z* 785 [M − H]^−^). Using the same approach characterized by the loss of sugar moieties, kaempferol-7-*O*-rutinoside (compound **14**, *m*/*z* 593 [M − H]^−^), keampferol-7-*O*-glucoside (compound **16**, *m*/*z* 447 [M − H]^−^), kaempferol-7-*O*-(6”-*O*-malonyl)-hexoside (compound **13**, *m*/*z* 533 [M − H]^−^), and kaempferol-3,7-di-*O*-hexoside (compound **12**, *m*/*z* 609 [M − H]^-^) were detected. Considering a parent ion with *m*/*z* 609, another isobaric compound was found in Moradyn extract (compound **10**); its fragmentation gave a base peak at *m/z* 301 [M – H − 308]^−^, and secondary peaks at *m*/*z* 300 (relative intensity 50%) and *m*/*z* 463 (relative intensity 5%), respectively. This fragmentation pattern could putatively be attributed to the presence of quercetin-7-*O*-rutinoside or more probably to quercetin-7-*O*-*p*-coumaroylhexoside, considering the very low abundance of *m*/*z* 463. Moreover, quercetin-7-*O*-glucoside (compound **11**, *m*/*z* 463 [M − H]^−^) was also identified and its chemical structure was confirmed by the analysis of the pure standard compound. Finally, myricetin-7-*O*-hexoside (compound **7**, *m*/*z* 479 [M − H]^−^) and myricetin-3,7-di-*O*-hexoside (compound **5**, *m/z* 641 [M − H]^−^) were detected, together with another compound with molecular mass 448 Da (compound **18**) whose MS^2^ spectrum gave as base peak an ion at *m*/*z* 285 and as secondary peaks the key aglycon fragments at *m*/*z* 241 and 175 [37]. The compound was identified as luteolin-7-*O*-glucoside (a flavone derivative) by comparing its fragmentation pattern with that of the reference standard compound. Another flavone derivative was present: compound **3** (*m*/*z* 431 [M − H]^−^), that fragmented to yield *m*/*z* 269 as the base peak ion and *m*/*z* 268 as the radical aglycon ion, thus leading to the identification of apigenin-7-*O*-glucoside; the analysis of the reference compound confirmed the chemical structure.

The last two compounds detected in the extract were hydroxycinnamic acid derivatives: compound **8** (*m*/*z* 367 [M − H]^−^) showed the typical pattern of 5-*O*-feruloylquinic acid [38], while compound **6** (*m*/*z* 459 [M − H]^−^) was a feruloyl derivative. Ferulic acid and its derivatives were previously detected in purple corn flour [39], in colored kernels from ten different genotypes [9], and in a particular Peruvian purple accession [40].

Anyway, even if the tested Moradyn phytocomplex derived from the morado variety, it is quite different from the other Peruvian varieties. In fact, no malonylated anthocyanins were detected; conversely, it is rich in flavonols such as quercetin, myricetin, isorhamnetin, and kaempferol derivatives. These differences are probably related not only to its genotype but also to environmental factors such as the photoperiod [1,8,21,39,40].

### 2.2. Evaluation of Hypoglycemic Activity

**Figure 2:**
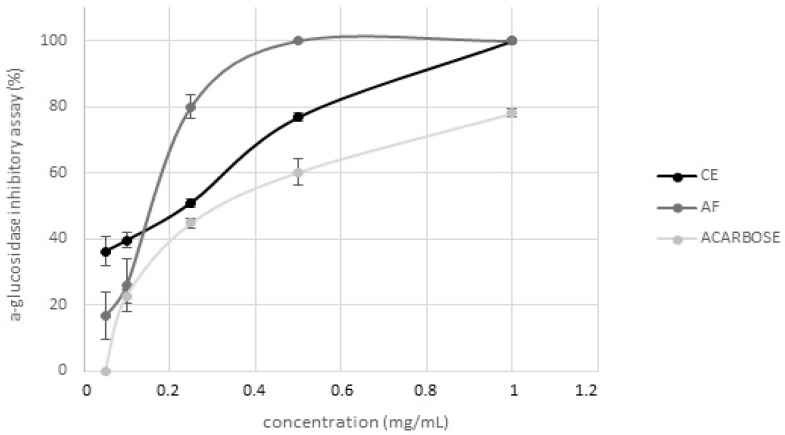
Evaluation of hypoglycemic activity of Moradyn CE and anthocyanin fraction (AF) by α-glucosidase inhibition assay; acarbose was used as positive control.

Hypoglycemic activity of CE and AF was tested by evaluating their inhibitory activity against α-glucosidase and α-amylase, two of the main enzymes involved in postprandial hyperglycemia modulation [41]. AF represents 28% (g/g dry CE) and this recovery agreed with the range of data reported in literature by Lee et al. [5] and Lao et al. [1] for other purple corn cob varieties. CE showed a strong dose-dependent inhibitory activity on α-glucosidase (Figure 2) which was completely inhibited in presence of 1 mg/mL CE, differently from acarbose (used as positive control) that inhibited only about 78% of the enzyme activity when tested at the same concentration. Regarding the isolated AF, it completely inhibited the enzyme activity when tested at 0.5 mg/mL. Conversely, neither CE nor AF were active against α-amylase at the same concentrations tested in the previous assay. The results here obtained are in agreement with those reported by Flores et al. [42] and Di Sotto et al. [43], indicating low inhibitory activity against α-amylase for extracts free of ellagitannins, syringic acid or rutin; moreover, flavonoids have a different effect on such enzymes depending on their conjugation site and class of the sugar moiety [44]. Even if cyanidin-3-*O*-glucoside, known to be a potent inhibitor [45], is the main anthocyanin present in CE, the simultaneous presence of many different polyphenols overall may lead to the loss of activity. Therefore, these results suggest that Moradyn hypoglycemic activity could be probably due to a strong synergistic action exerted by all the compounds present in the phytocomplex, and not only to the anthocyanin fraction.

### 2.3. Evaluation of Antiglycative Effect Using In Vitro Bovine Serum Albumin-Methylglyoxal, -Glucose, -Fructose, and -Ribose Systems

Several authors previously reported the antiglycative activity of different plant extracts mainly containing polyphenols [13,43,46]; in particular, anthocyanins showed a high effectiveness against protein glycation induced by monosaccharides and methylglyoxal (MGO) [47,48,49].

The antiglycative properties of CE and AF were investigated by evaluating the inhibition of AGEs formation in the middle and last stages of glycation by BSA-MGO and BSA-sugars assays, respectively.

**Figure 3:**
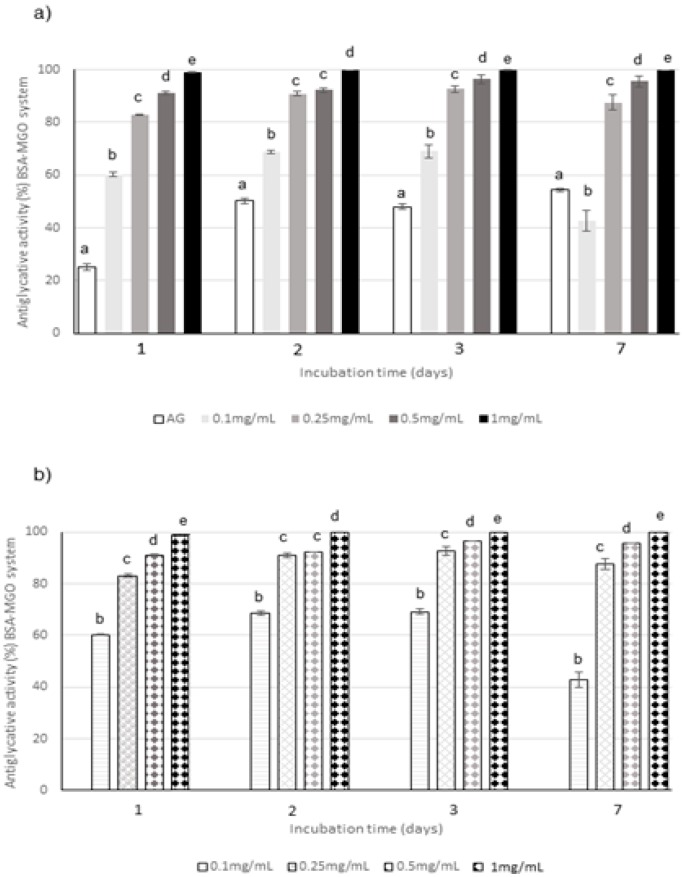
Antiglycative activity of Moradyn CE (**a**) and AF (**b**) on the formation of vesperlysine-like advanced glycation end products (AGEs) in BSA-MGO assay; AG, aminoguanidine (positive control). Different superscript letters within each monitoring time indicate significant differences (*p* < 0.05) among AG and CE or AG and AF at the different tested concentrations.

The capacity of the extract to act in the middle stage of protein glycation was evaluated in the system consisting of BSA and MGO; the inhibition of versperlysine-like AGEs formation at 0.1, 0.25, 0.5, and 1 mg/mL after 1, 2, 3, and 7 days of incubation was monitored. A dose-response relationship was registered for CE (Figure 3a). The phytocomplex tested at the highest concentration was able to completely prevent the AGEs formation starting from the beginning of the monitoring period and its action is always significantly higher than that of aminoguanidine (AG), used as positive control; conversely, the lowest concentration was able to inhibit only 40–60% of AGEs. The same trend was registered for AF when tested at the same concentrations and monitored at the same times. In fact, AF progressively increased its activity until 3 days when values higher than 90% were reached at all the concentrations with the exception of 0.1 mg/mL for which a decrease in the antiglycative capacity was registered at the end of the monitoring period (Figure 3b). Considering that the capacity registered for AF was generally similar to the one registered for the corresponding concentration of CE, and that AF represented only about 28% (*w*/*w*) of the complex, as previously mentioned, it is possible to conclude that AF highly contributed to the global antiglycative capacity in the BSA-MGO system.

**Figure 4:**
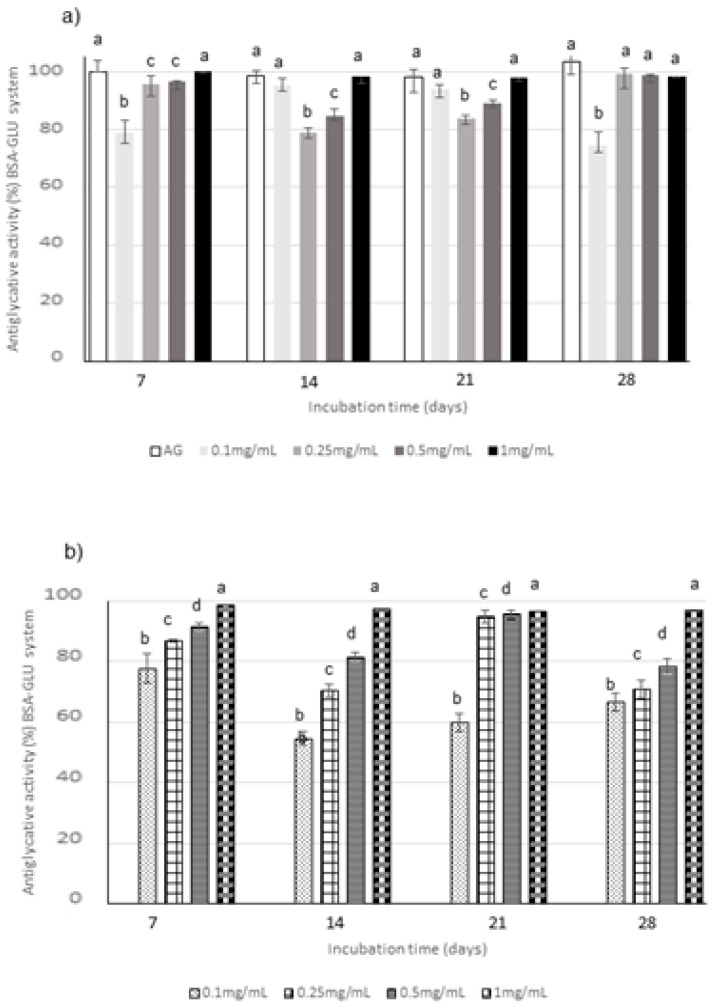
Antiglycative activity of Moradyn CE (**a**) and AF (**b**) on the formation of vesperlysine-like AGEs in BSA-GLU assay; AG, aminoguanidine (positive control). Different superscript letters within each monitoring time indicate significant differences (*p* < 0.05) among AG and CE or AG and AF at the different tested concentrations.

**Figure 5:**
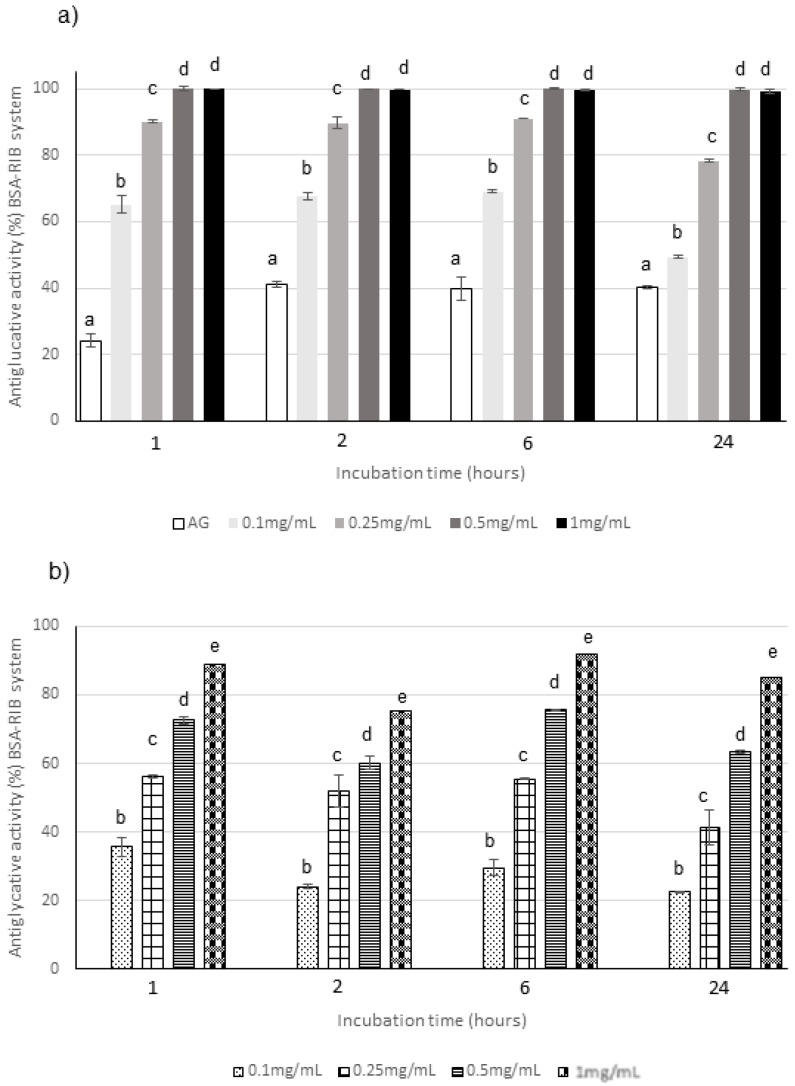
Antiglycative activity of Moradyn CE (**a**) and AF (**b**) on the formation of pentosidine-like AGEs in BSA-RIB assay; AG, aminoguanidine (positive control). Different superscript letters within each monitoring time indicate significant differences (*p* < 0.05) among AG and CE or AG and AF at the different tested concentrations.

**Figure 6:**
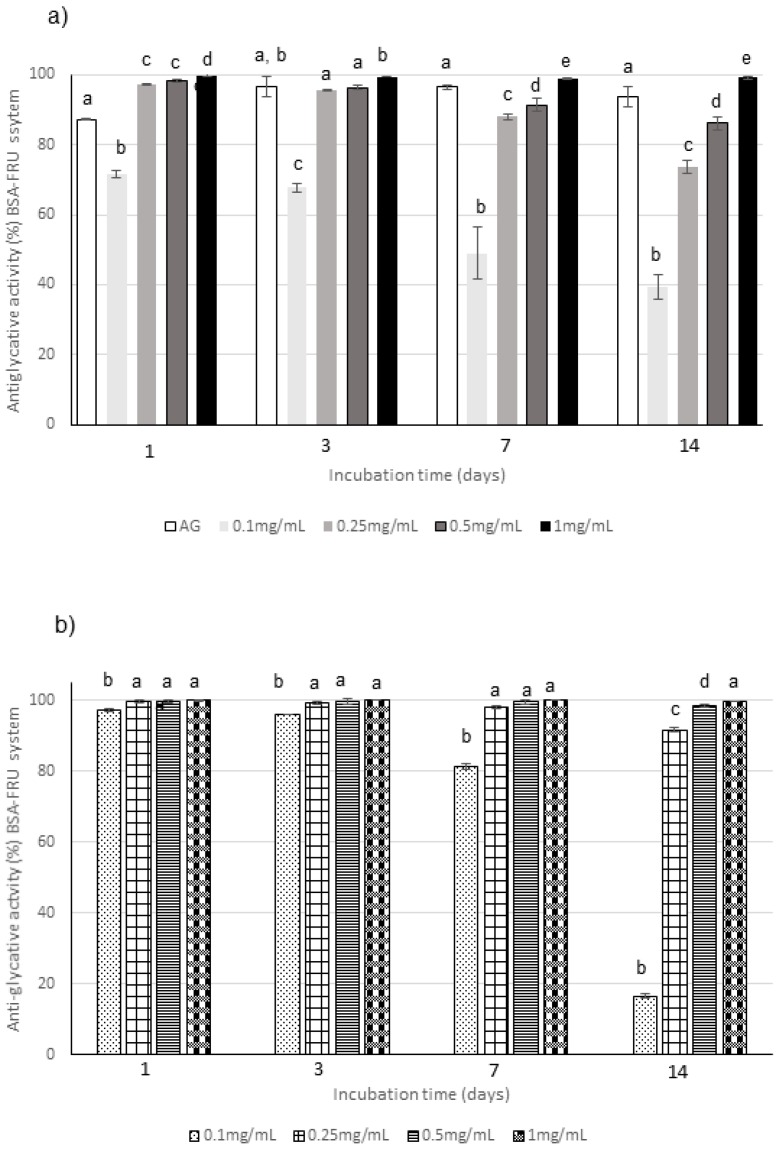
Antiglycative activity of Moradyn CE (**a**) and AF (**b**) on the formation of vesperlysine-like AGEs in BSA-FRU assay; AG, aminoguanidine (positive control). Different superscript letters within each monitoring time indicate significant differences (*p* < 0.05) among AG and CE or AG and AF at the different tested concentrations.

Regarding BSA-sugar systems, different monitoring times were selected according to the reactivity of each sugar: the glycation kinetics is slower for glucose (GLU), followed by fructose (FRU) and then by ribose (RIB), which is the most reactive sugar. Consequently, the inhibition of AGEs formation was monitored until 28 days, 14 days and 24 h for GLU, FRU, and RIB, respectively, according to the literature data [50]. The relationship between extract concentration and activity was evaluated through testing four different concentrations, i.e., 0.1, 0.25, 0.5, and 1 mg/mL, in all the BSA-sugar systems (Figure 4, Figure 5 and Figure 6). AG, known to be a potent antiglycative agent, was also used in these assays as positive control. AGEs formation is almost completely inhibited by 1 mg/mL CE in all the systems considered at all the monitoring times. Considering CE, the highest activity values were registered in the BSA-GLU system for all the tested concentrations with inhibitory values similar to those registered for AG (Figure 4a). In the BSA-RIB system, a dose-activity relationship was evident and the activity values were always higher than those registered for AG during the entire monitoring period (Figure 5a). A decrease in the AGEs inhibitory capacity was registered when FRU was present, especially for the lowest tested concentration (0.1 mg/mL) (Figure 6a).

The anthocyanin fraction had generally a lower capacity to inhibit AGEs formation in the system containing RIB, even if tested at the same concentration of the phytocomplex. In fact, it reached activity values of about 38% at 0.1 mg/mL and of 90% at 1 mg/mL (Figure 5b); a decrease in activity was also registered in the GLU system (Figure 4b). Conversely, very high capacity (always higher than 90% for 0.25–1 mg/mL) was detected in the system containing FRU during all the incubation time (Figure 6b).

According to Pearson’s correlation coefficient (R2), the correlation between α-glucosidase inhibitory activity and antiglycative activity tested with the different assays was always higher than 0.700 for CE over time during the monitoring period, with exception of the BSA-GLU system, highlighting that CE polyphenol composition is strongly related to both α-glucosidase inhibitory and antiglycative activities. Considering AF, higher R2 values were always registered probably due to a more marked dose-response relationship (Table 2).

Our antiglycative results are in agreement with the ones previously reported in the literature for berry extracts rich in polyphenols and especially in anthocyanins, which exhibited a strong AGEs inhibitory activity [48]. Moreover, several works also suggested that food byproducts rich in polyphenols are positively correlated with hypoglycemic [51] and antiglycative activities [10,51,52]. It is also well known that the different activity values registered for several food matrices and food byproducts are related to the qualitative and quantitative polyphenolic composition. In addition, different chemical structures of such compounds could highly affect the antiglycative activity [46], as confirmed by the results presented here, indicating a higher capacity to prevent AGEs formation for cob extract, which is rich in flavonoids and anthocyanins, than artichoke stem and outer bract extracts, which are rich in previously studied hydroxycinnamic acid derivatives [10].

### 2.4. Bioaccessibility Study

**Figure 7:**
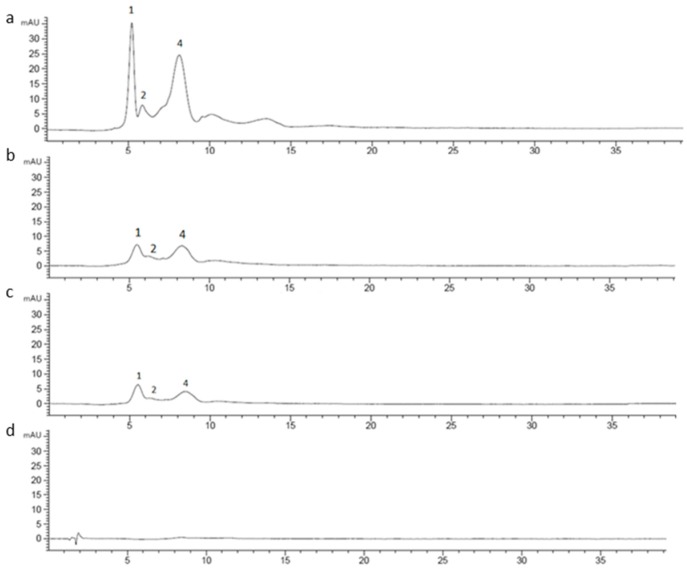
Chromatographic profile of CE (1 mg/mL) (**a**) undigested; (**b**) after oral phase; (**c**) after gastric phase; (**d**) after duodenal phase, registered at 520 nm.

**Figure 8:**
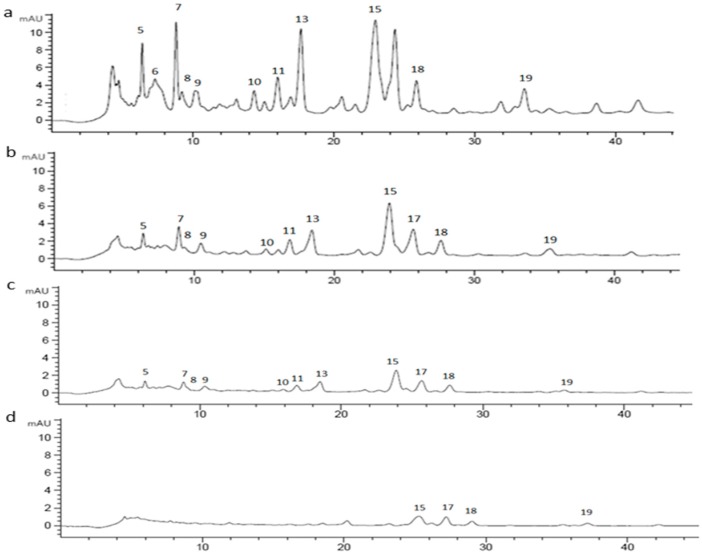
Chromatographic profile of CE (1 mg/mL) (**a**) undigested; (**b**) after oral phase; (**c**) after gastric phase; (**d**) after duodenal phase, registered at 370 nm.

CE (1 mg/mL) was digested using an in vitro static approach and the supernatants deriving from each step (oral, gastric, duodenal, and colon) of the process were analyzed by RP-HPLC-UV in order to investigate changes in the chromatographic profile. The chromatograms of undigested and digested CE were recorded both at 520 nm and 370 nm. Based on LC-MS^n^ results, 15 different marker compounds were selected in the undigested sample and monitored at each digestion step. In Table 3, the percentage of the relative peak area reduction for each marker was reported. A significant reduction was registered for all the monitored compounds (Figure 7 and Figure 8) mainly due to the gradual dilution of CE following the digestion steps (0.5 mg/mL, 0.25 mg/mL, 0.125 mg/mL, and 0.120 mg/mL in oral, gastric, duodenal, and colon phases, respectively). However, after the oral phase, compounds **4**, **6**, and **10** were present in lower amounts than expected, according to the dilution trend; for compounds **6** and **10**, this behavior could be due to a probable precipitation caused by a complex formation between compounds and enzyme [53,54,55]; for compound **4**, as previously reported by Xiao et al., the presence of a methoxy group on the B ring in peonidin-3-*O*-glucoside structure could strengthen the link between α-amylase and anthocyanin [56]. On the contrary, after the gastric step, cyanidin-3-*O*-glucoside and perlagonidin-3-*O*-glucoside relative abundances were higher than expected. This observation agreed with data already reported in the literature, according to which the strong acidic pH of the gastric phase and the presence of pepsin caused the anthocyanin bounded fraction release, thus increasing the concentration of each molecule [57,58]. Significant changes in the profile were registered after the duodenal step (Figure 7 and Figure 8) for all the peak markers; in particular, the peak area of each anthocyanin was approximately zero, confirming their high instability at basic pH [57,59]. In the colon phase the results were quite similar to those obtained in the duodenal phase (as evident in Table 3), and for this reason the chromatograms of the colon step were not reported in Figure 7 and Figure 8. The above described results agreed with data reported by Chang et al. [60] and by Tavares et al. [61], who assessed the bioaccessibility of cranberry bean and blackberry polyphenols, respectively. In fact, both these works reported an increase in the concentration of the polyphenolic compounds after the gastric phase and a subsequent strong reduction at the end of the intestinal phase, probably due to the interactions of polyphenols with proteins, generally occurring at neutral or basic pH [60].

**Figure 9:**
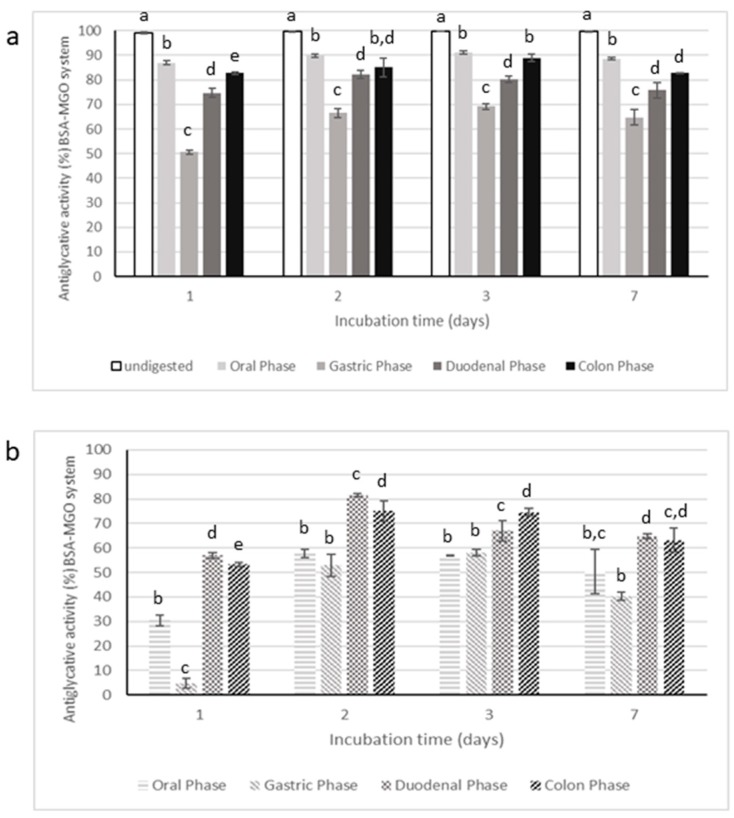
Antiglycative activity of digested Moradyn CE (**a**) and its negative control (**b**) on the formation of vesperlysine-like AGEs in BSA-MGO assay. Different superscript letters within each monitoring time indicate significant differences (*p* < 0.05) among undigested and digested CE or undigested CE and digested negative control.

**Figure 10:**
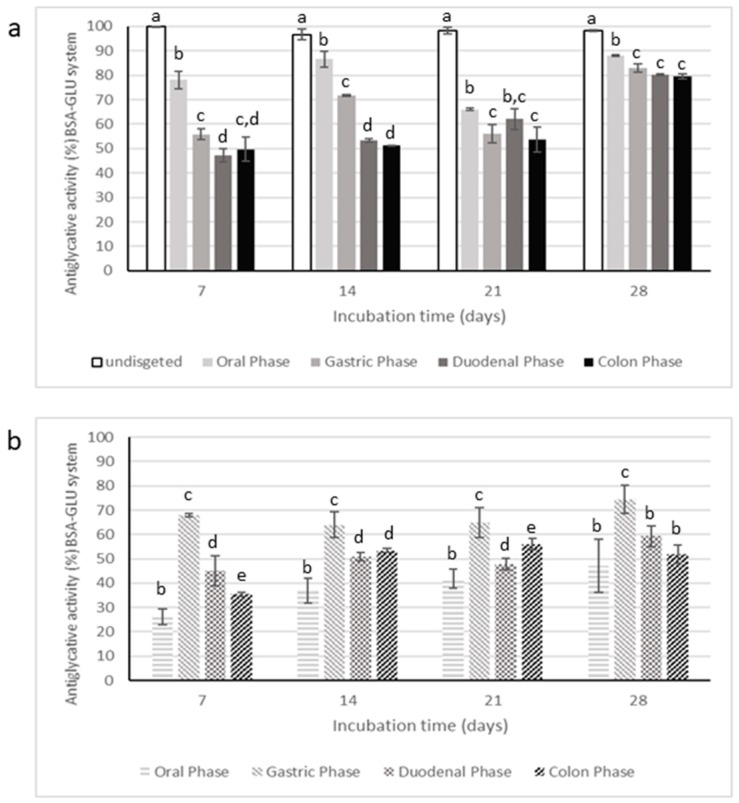
Antiglycative activity of digested Moradyn CE (**a**) and its negative control (**b**) on the formation of vesperlysine-like AGEs in BSA-GLU assay. Different superscript letters within each monitoring time indicate significant differences (*p* < 0.05) among undigested and digested CE or undigested CE and digested negative control.

Antiglycative activity of digested samples were evaluated only in BSA-MGO and BSA-GLU systems, considered as the most representative tests. The data are reported in Figure 9 and Figure 10, respectively.

Digestion strongly affected the inhibitory activity of CE on AGEs formation, starting from the oral phase; in fact, the supernatants collected after this step had significantly lower antiglycative capacity than the undigested CE in BSA-MGO and BSA-GLU systems. The gastric step reduced about 50% of the inhibitory activity after 1 day of incubation in the BSA-MGO system and then this activity progressively increased over the monitoring time (2, 3, and 7 days). Finally, CE activity appeared quite totally recovered after the duodenal and colon phases. This trend was also reported for the negative control, consisting of an appropriate mixture of digestive fluids and enzymes for each phase. Thus, it is possible to speculate that low AGEs formation recorded for CE after the duodenal and colon phases could be due to interactions occurring between MGO and digestive enzymes, leading to non-fluorescent AGEs formations and, therefore, to false positive results.

In the BSA-GLU system, the oral phase did not highly affect the antiglycative capacity. On the contrary, the gastric phase caused a reduction in the inhibitory activity down to about 55%; the following intestinal steps only slightly affected the activity. Nevertheless, considering the inhibitory capacity of the negative control, the antiglycative action of CE after both gastric and intestinal phases could be mainly associated with interaction between GLU and digestive enzymes, as in the MGO system. Therefore, it is possible to conclude that CE was still effective only after the oral phase.

Hypoglycemic activity of digested CE was also tested. Inhibitory values registered against α-glucosidase (24.61% ± 1.19%, 5.24% ± 2.43%, 0.97% ± 1.17 % 1.37% ± 2.38% for oral, gastric, duodenal, and colon phase, respectively) highly decreased step by step until the end of the process when CE had no activity. Therefore, digestion seemed to strongly compromise CE hypoglycemic activity as demonstrated by the consistent gradual decrease in the α-glucosidase inhibitory capacity occurring during the process. This trend could not be attributed only to the reduction in anthocyanin content, but rather to the decrease in the total polyphenol content. This result confirmed that CE hypoglycemic action should be ascribed to the synergistic action of all compounds present in the phytocomplex, as already explained and shown in Figure 2.

## 3. Materials and Methods

### 3.1. Reagents

Ethanol, methanol, d-(+)-glucose (GLU), d-(−)-fructose (FRU), disodium hydrogen phosphate dodecahydrate, sodium carbonate decahydrate and sodium bicarbonate were purchased from Carlo Erba (Milano, Italy). HPLC-MS-grade formic acid and acetonitrile, hydrochloric acid (37%), Type VI-porcine pancreatic α-amylase, α-glucosidase from Saccharomices cerevisiae, pepsin from porcine gastric mucosa (≥400 U mg^−1^), bile extract porcine, pancreatin (8× USP) from porcine pancreas, protease from Streptomyces griseus type XIV (≥3.5 U mg^−1^), and viscozyme L cellulolytic enzyme mixture, *p*-nitrophenyl-alpha-d-glucopyranoside (purity grade ≥ 99%), starch, acarbose (purity grade ≥ 95%), sodium potassium tartrate tetrahydrate, dinitrosalicylic acid (DNS), methylglyoxal (MGO, 40% aqueous solution), aminoguanidine hydrochloride (AG, purity grade ≥ 98%), bovine serum albumin (BSA, purity grade ≥ 98%), d-(−)-ribose (RIB, purity grade ≥ 98%), sodium dihydrogen phosphate monohydrate, disodium hydrogen phosphate monohydrate, sodium hydroxide pellets, sodium chloride, sodium azide (purity grade 99.5%), pelargonidin-3-*O*-glucoside, apigenin-7-*O*-glucoside, and quercetin-7-*O*-glucoside were provided by Merck KGaA (Darmstadt, Germany).

Water was obtained from a Millipore Direct-QTM system (Merck-Millipore, Milan, Italy). Kuromanin chloride (cyanidin-3-*O*-glucoside), peonidin-3-*O*-glucoside, kaempferol-7-*O*-glucoside, and luteolin-7-*O*-glucoside were purchased from Extrasynthese (Genay, Rhone, France).

Sep-Pak C18 cartridges (6 mL, 1 g sorbent) were purchased from Waters Corporation (Massachussets, MA, USA).

### 3.2. Moradyn Cob Extract Preparation

The Moradyn population has been developed in Italy (Lombardy region, 2015–2018) starting from a cross hybrid morado variety (South America) by a pedigree selection of photoperiod, seed, and cob pigmentation. This variety has been submitted to the Community Plant Variety Office for the registration (Examination Ref. 4067062) by FlaNat Research Italia Srl. Moradyn cobs were chopped into small pieces (about 1–2 cm) and extracted by FlaNat Research Italia S.r.l. (Milan, Italy) with aqueous ethanol for 3 h at 50 °C. The resulting suspension was filtered through 0.45 µm membrane filters (Merck-Millipore, Milan, Italy) and after removing organic solvent under reduced pressure at 40 °C (Buchi R-II, Buchi, UK), the cob extract (CE) was freeze-dried (Modulyo freeze-drier s/n 5101, Akribis scientific limited, Cheshire, UK ) and then used in the experiments.

### 3.3. Anthocyanin Fraction Purification

Anthocyanin fraction (AF) isolation was performed using a Sep-Pak C18 cartridge, following the procedure reported by Scorrano et al. [62] with some modifications. The cartridge was activated with 10 mL of methanol followed by 20 mL of acidified water (0.01% *v*/*v* HCl). Then, 5 mL of ten-fold concentrated extract was loaded. After having removed sugars and phenolic acids with 10 mL of acidified water and less polar compounds with 10 mL of ethyl acetate, AF was collected with 10 mL of acidified methanol (0.01% *v*/*v* HCl). The recovered AF was evaporated to remove solvent and then freeze-dried, obtaining a ready-to-use powder to be opportunely re-suspended for each assay.

### 3.4. RP-HPLC-DAD-ESI-MS^n^ Analysis

Separation and identification of compounds present in the phytocomplex were performed using a Thermo Finningan Surveyor Plus HPLC apparatus (Thermo Fischer Scientific, Waltham, MA, USA) equipped with a quaternary pump, a Surveyor UV-Vis photodiode-array detector, a Surveyor Plus autosampler, and a vacuum degasser connected to a LCQ Advantage Max ion trap spectrometer through an ESI source. A Gemini^®^ C18 analytical column (150 × 2.0 mm i.d., 5 μm, Phenomenex, Torrance, CA, USA) operating at a constant flow rate of 0.3 mL/min (injection volume 20 μL) was used for the separation. The mobile phase consisted of 0.1% formic acid aqueous solution (solvent A) and of 0.1% formic acid in acetonitrile (solvent B) with the following gradient table: 0–3 min, 2–15% B; 3–45 min, 15–25% B; 45–48 min, 25–35% B; 48–58 min, 2% B, column reconditioning: 10 min. UV-Vis spectral data of samples were acquired in the range 200–700 nm, and chromatograms were recorded at 280, 325, 370, and 520 nm.

The parameters of the ion mode ESI source had previously been optimized to a ionization voltage of 5 kV, a capillary voltage of +36 V, a capillary temperature of 300 °C, a sheath gas flow rate of 20 arbitrary units (AU), and an auxiliary gas flow rate of 10 AU. The Thermo Fisher Scientific Excalibur 2.2 software was used for data acquisition and processing. The ion trap operated in data-dependent, full scan (60–2000 *m*/*z*), and MS^n^ mode to obtain fragment ion *m*/*z* with a 35% and an isolation width of 3 *m*/*z*. Three independent assays were performed. Identification of individual phenolic compounds was carried out by comparing their retention times, UV-Vis spectra, and MS patterns of fragmentation with those obtained for original standards, when commercially available.

### 3.5. Evaluation of Hypoglycemic Activity

Both CE and AF were tested at different concentrations (ranging from 50 µg/mL to 1 mg/mL) to evaluate their ability to inhibit α-amylase and α-glucosidase activities. CE digestion fractions were tested only for α-amylase inhibitory activity. Data were expressed as the means ± standard deviation (SD) of four independent experiments for each tested concentration. They were considered statistically significant with *p* values <0.05.

#### 3.5.1. α-Amylase Inhibitory Activity

The α-amylase inhibition assay was performed following the method reported by Milella et al. [41] with some modifications. Briefly, 500 μL of Type VI-B porcine pancreatic α-amylase (0.5 mg/mL in 100 mM sodium phosphate buffer, containing 6.7 mM sodium chloride, pH 6.7-PBS) were preincubated with 500 μL of each sample (CE or AF) in test tubes at 37 °C for 10 min. Then, 500 μL of 1% starch (*w*/*v*, previously suspended in 100 mM PBS, pH 6.7, and boiled for 10 min) was added. This mixture was incubated for 10 min. Finally, 1 mL of DNS reagent (consisting of 20 mL of 96 mM DNS, 8 mL of 5.315 M sodium potassium tartrate tetrahydrate in 2 M NaOH and 12 mL of distilled water) was added. The tubes were placed in a boiling water bath (5 min) to stop the reaction, then cooled at room temperature and diluted with 10 mL of distilled water. DNS reacted with free reducing sugars released by enzymatic hydrolysis of starch, leading to a change in the color of the sample (from yellow to deep red). Absorbance was read at 520 nm. In order to remove the background signals, the absorbances of all samples were also registered in absence of the enzyme solution. Sample made of only starch and enzyme solution were used as a negative control while acarbose solutions (ranging from 5 µg/mL to 100 µg/mL) were used as a positive control.

The results were expressed as α-amilase inhibition percentage, calculated using the following formula:

Inhibition rate (%) = [1 − (Abs 520 nm sample – Abs 520 nm background/Abs 520 nm negative control)] × 100

#### 3.5.2. α-Glucosidase Inhibitory Activity

The capacity of the tested samples to inhibit α-glucosidase activity was performed according to method proposed by Flores et al. [42] with slight modifications. A total of 100 μL of α-glucosidase solution (0.35 U/mL 100 mM PBS, pH 6.7) was incubated with 50 µL of sample solution for 10 min at 37 °C in a test tube. Then, 100 µL of 1.5 mM p-nitrophenyl-α-d-glucopyranoside solution (an artificial substrate which released a yellowish phenolic compound when hydrolyzed by α-glucosidase) was added and the final mixture was left for 20 min at 37 °C. The reaction was stopped by adding 1 mL 1 M sodium carbonate decahydrate and the absorbance was read at 400 nm.

Samples without the enzyme solution were used as blank. A mixture containing all reactants was used as a negative control. Acarbose solutions (0.05–1mg/mL) were used as a positive control.

The results were expressed as α-glucosidase inhibition percentage, calculated using the following formula:

Inhibition rate (%) = [1 − (Abs 400 nm mixture containing sample – Abs 400 nm blank/Abs 400 nm negative control)] × 100

### 3.6. Evaluation of Antiglycative Capacity

The antiglycative properties of both CE and its AF were evaluated by using three different in vitro BSA-sugars systems (glucose, GLU; ribose, RIB; fructose, FRU) and a BSA-MGO system, following the methods proposed by Mesìas et al. [11] and by Maietta et al. [63], slightly modified. In all systems, AG was used as positive control. Conversely, digested CE supernatants were tested only in BSA-GLU and BSA-MGO assays.

Stock solutions were prepared by dissolving BSA (35 mg/mL), GLU (175 mg/mL), RIB (150 mg/mL), FRU (175 mg/mL), MGO (0.4 mg/mL), and AG (0.5 mg/mL) in 0.1 M phosphate buffer (pH 7.4) containing 0.02% (*w*/*v*) of sodium azide (to avoid microbial contamination). Sample solutions were prepared by dissolving freeze-dried material in a proper volume of phosphate buffer to obtain 0.1, 0.25, and 1 mg/mL final concentrations in the reaction mixtures. Then, 400 μL of BSA solution, 800 μL of sugar solution, and 300 μL of sample solution or phosphate buffer (blank) were mixed and the final reaction mixture was subsequently incubated at 37 °C in a thermostatted bath (Memmert basic WNB, Schwabach, Germany).

The systems were incubated for different times depending on sugars and MGO reactivity [64], as follows: GLU systems were incubated for 28 days, monitoring AGEs formation once a week, while systems containing MGO and FRU for 7 and 14 days, respectively, with analytical determination at 1, 3, 7, and 14 days. Samples containing RIB were incubated for 24 h evaluating the AGEs formation at 1, 3, 6, and 24 h. At the end of each monitored time, the reaction was stopped in an ice bath (15 min) before the analysis. Vesperlysine-like (λ_exc_ 370 nm, λ_em_ 440 nm) or Pentosidine-like (λ_exc_ 335 nm; λ_em_ 420 nm) fluorescent AGEs (Spectrofluorometer Perkin Elmer L550B) were monitored for BSA-GLU, BSA-FRU, and BSA-MGO systems, or for BSA-RIB, respectively. CE and AF phosphate buffer solutions were incubated at the same reaction times to evaluate their intrinsic fluorescence.

The inhibition of AGE formation (%) was calculated using the equation below:

Inhibition % = [1 − (Fluorescence of incubation mixture containing sample – Intrinsic fluorescence of sample/Fluorescence of incubation mixture without sample)] × 100

The reported data are the means ± standard deviation (SD) of three independent experiments for each tested concentration, each performed in triplicate. They were considered statistically significant with *p* values of <0.05.

### 3.7. In Vitro Digestion Procedure

The in vitro gastrointestinal digestion model was designed following the INFOGEST protocol [65], with some modifications; briefly, simulated salivary fluid (SSF), simulated gastric fluid (SGF), and simulated intestinal fluid (SIF) were basically prepared with electrolytes, enzymes, bile salts, and water; pepsin and pancreatin were added to SGF and SIF, respectively, while the colon phase was simulated according to Hamzalıoğlu procedure [66]. At the end of each step, enzymes were inactivated (90 °C, 5 min) and digestion mixtures were centrifuged (30 min, 4 °C, 5000 rpm, Centrifuge 5804 R Eppendorf). Supernatants were freeze-dried and stored at −20 °C until analyses.

A total of 1 mg/mL aqueous CE solution was digested, while the negative control consisted of an aqueous solution of digestion fluids and enzymes submitted to the same four-step procedure.

### 3.8. Bioaccessibility Evaluation

The percentage of soluble polyphenols collected at the end of each digestion step represents the CE fraction available for absorption [29,58].

To monitor the gastrointestinal effects on CE composition, the oral, gastric, duodenal, and colon fractions were analyzed using an Agilent Technologies 1260 Infinity II technology series system (Santa Clara, CA, USA), equipped with quaternary gradient pump, a vial sampler, a degasser, a thermostatted column oven set at 25.0 ± 0.5 °C, and a variable wavelength detector (VWD). The HPLC-VWD system was controlled using a personal computer equipped with Agilent OpenLab CDS ChemStation software on Windows 10. Separation was carried out on the same column and using the same gradient elution reported for RP-HPLC-DAD-ESI-MS^n^ analysis (Section 3.4). Chromatograms were recorded at 520 nm and 370 nm.

Furthermore, supernatants obtained by each digestion step were also tested for their antiglycative capacity using BSA-MGO and BSA-GLU systems and for hypoglycemic activity, assessed as α-glucosidase inhibitory activity.

### 3.9. Statistical Analysis

Statistical analysis of the data and Pearson’s correlation coefficients were performed using Microsoft Office 365. The significant differences (*p* < 0.05) were evaluated by variance analysis (ANOVA).

## 4. Conclusions

The reuse of agro-industrial byproducts represents a very important issue in the circular bio-economy and a potential resource of new products and compounds. Moradyn is a new Italian purple corn variety rich in polyphenols, including anthocyanin derivatives, such as cyanidin-3-*O*-glucoside, perlagonidin-3-*O*-glucoside, and peonidin-3-*O*-glucoside. It was demonstrated to possess a good capacity to inhibit α-glucosidase in an *in vitro* system and therefore it could act in blocking the intestinal carbohydrate-digesting enzyme activity.

Moreover, Moradyn phytocomplex could prevent AGEs formation, acting with a strong antiglycative agent as demonstrated in this work using in vitro model systems. The registered healthy effects were not only due to anthocyanin fraction, but more probably to a synergistic action of all polyphenols present in the extract. A further step consisting of the evaluation of bioaccessibility of Moradyn CE indicated that the in vitro digestion process caused a marked decrease in the tested bioactivities. These results highlight that such studies are mandatory to obtain preliminary information useful for proceeding with the selection of suitable coating agents in order to preserve bioactivities. Moreover, once the carrier has been selected, stability studies on the final ingredient to be marketed and efficacy on glucose uptake and adipogenesis using cell-based assays will be performed.

## Figures and Tables

**Table 1:** MS and MS^n^ data (negative or positive ionization modes) of the compounds identified in Moradyn cob extract (CE). Compounds are reported in order of elution; * positive ionization mode; ^a^ compared with standard compounds.

Compound	Rt (min)	Precursor Ion (*m*/*z*)	HPLC-ESI-MS^n^ *m*/*z* (% of Base Peak)	Compound Identity
**1** ^a^	5.92	449 *	MS2[449]: 287(100)	cyanidin-3-*O*-glucoside
**2** ^a^	6.41	433 *	MS2[433]: 271(100)	pelargonidin-3-*O*-glucoside
**3** ^a^	6.53	431	MS2[431]: 269 (100), 268 (85)	apigenin-7-*O*-glucoside
**4** ^a^	8.23	463 *	MS2[463]: 301(100)	peonidin-3-*O*-glucoside
**5**	8.49	641	MS2[641]: 479(100), 317(27) MS3[479]: 317(100)	myricetin-3,7-di-*O*-hexoside
**6**	8.70	459	MS2[459]: 235(30), 193(100), 149(30)	ferulic acid derivative
**7**	9.77	479	MS2[479]: 317(100), 316(5), 299(70)	myricetin-7-*O*-hexoside
**8**	10.67	367	MS2[367]: 191(100), 173(20) MS3[191]: 172(40), 127(80), 85(100)	5-*O*-feruloylquinic acid
**9**	11.00	639	MS2[639]: 477(100), 315(5) MS3[477]: 315(100), 300(10)	isorhamnetin-3,7-di-*O*-hexoside
**10**	15.76	609	MS2[609]: 463(5), 301(100), 300(50)	quercetin-7-*O*-*p*-cumaroylhexoside
**11** ^a^	17.17	463	MS2[463]: 301(100), 300(30)	quercetin-7-*O*-glucoside
**12**	17.97	609	MS2[609]: 447(100), 285(30)	keampferol-3,7-di-*O*-hexoside
**13**	19.97	533	MS2[533]: 447(26), 285(100), 284(38) MS3[285]: 267(15), 257(100), 241(25), 199(10), 163(5)	keampferol-7-O-(6”-O-malonyl)-hexoside
**14**	20.66	593	MS2[593]: 447(70), 285(100), 257(15) MS3[285]: 267(15), 257(100), 241(10), 199(20)	keampferol-7-*O*-rutinoside
**15**	21.85	623	MS2[623]: 477(20), 315(100) MS3[315]: 300(100)	isorhamnetin-7-*O*-rutinoside
**16**	22.24	447	MS2[447]: 327(20), 285(100), 284(85), 257(30), 255(5)	kaempferol-7-*O*-glucoside
**17**	23.26	477	MS2[477]: 315(35), 314(100) MS3[315]: 300(100)	isorhamnetin-3-*O*-hexoside
**18** ^a^	27.52	447	MS2[447]: 285(100), 284(95), 151(10), 133(6) MS3[285]: 267(40), 257(20), 241(40), 199(10), 175(100)	luteolin-7-*O*-glucoside
**19**	33.87	785	MS2[785]: 609(20), 447(100) MS3[447]: 285(100), 284(80)	kaempferol-3-*O*-hexosyl-7-*O*-glucuronilhexoside
**20**	34.67	815	MS2[815]: 639(60), 477(100) MS3[477]: 315(100), 300(10)	isorhamentin-3-*O*-hexosyl-7-*O*-glucuronilhexoside

**Table 2:** Pearson’s correlation coefficients (R2) among α-glucosidase inhibitory activity and antiglycative activities monitored at different times of Moradyn CE and AF.

Assay	CE α-Glucosidase Inhibitory Activity	AF α-Glucosidase Inhibitory Activity
BSA-MGO system		
1 day	0.908	0.977
2 days	0.840	0.971
3 days	0.813	0.991
7 days	0.800	0.993
BSA-GLU system		
7 day	0.802	0.885
14 days	0.337	0.895
21 days	0.488	0.970
28 days	0.021	0.718
BSA-FRU system		
1 day	0.728	0.984
4 days	0.745	0.987
7 days	0.803	0.982
14 days	0.915	0.983
BSA-RIB system		
1 h	0.826	0.918
3 h	0.841	0.954
6 h	0.827	0.935
24 h	0.880	0.879

**Table 3:** Relative peak area reduction (%) of CE selected marker compounds after each simulated gastrointestinal digestion step (oral, gastric, duodenal, and colon).

Compound	Oral Phase	Gastric Phase	Duodenal Phase	Colon Phase
**1**	63.96 ± 1.4	69.78 ± 2.60	100	100
**2**	57.86 ± 0.73	73.33 ± 1.18	100	100
**4**	74.94 ± 0.78	84.45 ± 1.94	100	100
**5**	61.04 ± 0.48	80.03 ± 0.24	100	100
**6**	100	100	100	100
**7**	54.97 ± 0.69	84.54 ± 0.68	100	100
**8**	58.96 ± 0.87	84.95 ± 0.26	100	100
**9**	48.21 ± 5.37	72.37 ± 1.12	95.94 ± 0.15	100
**10**	70.08 ± 0.10	94.70 ± 0.90	100	100
**11**	52.85 ± 0.05	79.07 ± 0.25	94.44 ± 0.37	98.67 ± 0.32
**13**	56.46 ± 0.79	82.78 ± 0.69	93.70 ± 0.44	98.9 ± 0.80
**15**	50.64 ± 1.64	80.67 ± 0.22	97.95 ± 0.45	94.99 ± 0.37
**17**	66.64 ± 1.30	86.17 ± 0.14	90.54 ± 0.24	95.4 ± 0.24
**18**	45.78 ± 1.84	73.44 ± 0.92	83.26 ± 0.23	86.46 ± 0.20
**19**	60.53 ± 2.02	89.44 ± 0.16	100	100
